# The hydraulic efficiency–safety trade‐off differs between lianas and trees

**DOI:** 10.1002/ecy.2666

**Published:** 2019-04-08

**Authors:** Masha T. van der Sande, Lourens Poorter, Stefan A. Schnitzer, Bettina M. J. Engelbrecht, Lars Markesteijn

**Affiliations:** ^1^ Department of Community Ecology Helmholtz Centre for Environmental Research – UFZ Theodor‐Lieser‐Straße 4 06120 Halle (Saale) Germany; ^2^ German Centre for Integrative Biodiversity Research (iDiv) Halle‐Jena‐Leipzig Deutscher Platz 5e 04103 Leipzig Germany; ^3^ Forest Ecology and Forest Management Group Wageningen University and Research P.O. Box 47 6700 AA Wageningen The Netherlands; ^4^ Department of Biological Sciences Florida Institute of Technology Melbourne Florida FL 32901 USA; ^5^ Institute for Biodiversity and Ecosystem Dynamics University of Amsterdam Amsterdam The Netherlands; ^6^ Department of Biological Sciences Marquette University PO Box 1881 Milwaukee Wisconsin 53201 USA; ^7^ Smithsonian Tropical Research Institute Apartado 0843‐03092 Balboa Ancon Republic of Panama; ^8^ Department of Plant Ecology Bayreuth Center of Ecology and Environmental Research (BayCEER) University of Bayreuth 95440 Bayreuth Germany; ^9^ School of Natural Sciences Bangor University Bangor Gwynedd LL57 2DG United Kingdom

**Keywords:** drought tolerance, functional traits, hydraulic architecture, hydraulic conductivity, lianas, *P*_50_, Panama, plant–water relations, species abundance, tropical forest

## Abstract

Hydraulic traits are important for woody plant functioning and distribution. Associations among hydraulic traits, other leaf and stem traits, and species’ performance are relatively well understood for trees, but remain poorly studied for lianas. We evaluated the coordination among hydraulic efficiency (i.e., maximum hydraulic conductivity), hydraulic safety (i.e., cavitation resistance), a suite of eight morphological and physiological traits, and species’ abundances for saplings of 24 liana species and 27 tree species in wet tropical forests in Panama. Trees showed a strong trade‐off between hydraulic efficiency and hydraulic safety, whereas efficiency and safety were decoupled in lianas. Hydraulic efficiency was strongly and similarly correlated with acquisitive traits for lianas and trees (e.g., positively with gas exchange rates and negatively with wood density). Hydraulic safety, however, showed no correlations with other traits in lianas, but with several in trees (e.g., positively with leaf dry matter content and wood density and negatively with gas exchange rates), indicating that in lianas hydraulic efficiency is an anchor trait because it is correlated with many other traits, while in trees both efficiency and safety are anchor traits. Traits related to shade tolerance (e.g., low specific leaf area and high wood density) were associated with high local tree sapling abundance, but not with liana abundance. Our results suggest that different, yet unknown mechanisms determine hydraulic safety and local‐scale abundance for lianas compared to trees. For trees, the trade‐off between efficiency and safety will provide less possibilities for ecological strategies. For lianas, however, the uncoupling of efficiency and safety could allow them to have high hydraulic efficiency, and hence high growth rates, without compromising resistance to cavitation under drought, thus allowing them to thrive and outperform trees under drier conditions.

## Introduction

Hydraulic efficiency, safety, and associated traits are important in determining tree species’ functioning (Sterck et al. 2011, Cosme et al. 2017) and response to drought (Rowland et al. 2015, Anderegg et al. 2016) and may therefore be important for predicting future shifts in tree species’ performance and distribution (Anderegg et al. 2012). We know, however, little about hydraulic traits and their relationships with other traits and performance for lianas. Understanding associations and trade‐offs among such traits, and whether these differ between lianas and trees, will enhance our understanding of growth strategies, functioning and distribution of liana and tree species.

High hydraulic efficiency (e.g., high sapwood‐specific maximum hydraulic conductivity) is associated with high photosynthetic efficiency (Brodribb et al. 2004, Santiago et al. 2004) and, hence, allows species to grow rapidly (Poorter 1999), especially in moist, high‐light environments. However, tissue investments that enhance hydraulic efficiency, such as wide and long vessels that are associated with low wood density, usually decrease hydraulic safety (Lens et al. 2011, Markesteijn et al. 2011b), which may be one of the factors preventing these species from occurring in dry areas. There is mixed support for a trade‐off between hydraulic efficiency and safety for trees, with some studies showing a trade‐off (Hacke et al. 2006, Markesteijn et al. 2011b, De Guzman et al. 2016) where others do not (Gleason et al. 2015, Santiago et al. 2018). However, empirical comparisons of the trade‐offs in functional traits between lianas and trees are not common. Lianas generally have lower hydraulic safety and wood density due to less investment in supporting stem tissues, and higher hydraulic efficiency (Zhu and Cao 2009, van der Sande et al. 2013, De Guzman et al. 2016). If, for lianas, hydraulic efficiency is not constrained by hydraulic safety (i.e., no trade‐off), then lianas could have high conductivity and remain photosynthetically active without being very vulnerable in dry conditions, which could then contribute to explaining why lianas tend to become relatively more abundant toward seasonal forests (Schnitzer 2005). To our knowledge, only one study has assessed the relationship between efficiency and safety for lianas and trees. De Guzman et al. (2016) found a trade‐off between hydraulic efficiency and safety among six liana and six tree species in a seasonally dry tropical forest in Panama, which appeared similar for lianas and trees (Santiago et al. 2015). However, they did not formally test for differences between lianas and trees in the efficiency–safety trade‐off.

Apart from the trade‐off between hydraulic efficiency and hydraulic safety, several studies have shown that physical and ecological limitations can also impose trade‐offs and synergies of hydraulic traits with other traits. For example, species with high hydraulic efficiency also have high gas exchange rates, which contributes to an acquisitive life history strategy of fast resource acquisition, growth, and tissue turnover (Santiago et al. 2004). Hence, hydraulic efficiency may be positively associated with traits related to an acquisitive growth strategy (photosynthetic efficiency, stomatal conductance, specific leaf area) and transporting tissue morphology (maximum vessel length). Species with high hydraulic safety, however, have low gas exchange rates and other traits related to a conservative life history strategy of resource conservation and slow growth and tissue turnover (Markesteijn et al. 2011b). Hydraulic safety may therefore be associated with traits that comprise a conservative growth strategy (high leaf dry matter content, wood density, water use efficiency, and Huber value). Although liana and tree seedlings have generally similar life‐history trade‐offs (Gilbert et al. 2006), they may differ in associations of hydraulic traits with other morphological and physiological traits such as wood density, vessel length, and photosynthetic efficiency, and in the relationship between traits and their local abundance.

**Figure 1:**
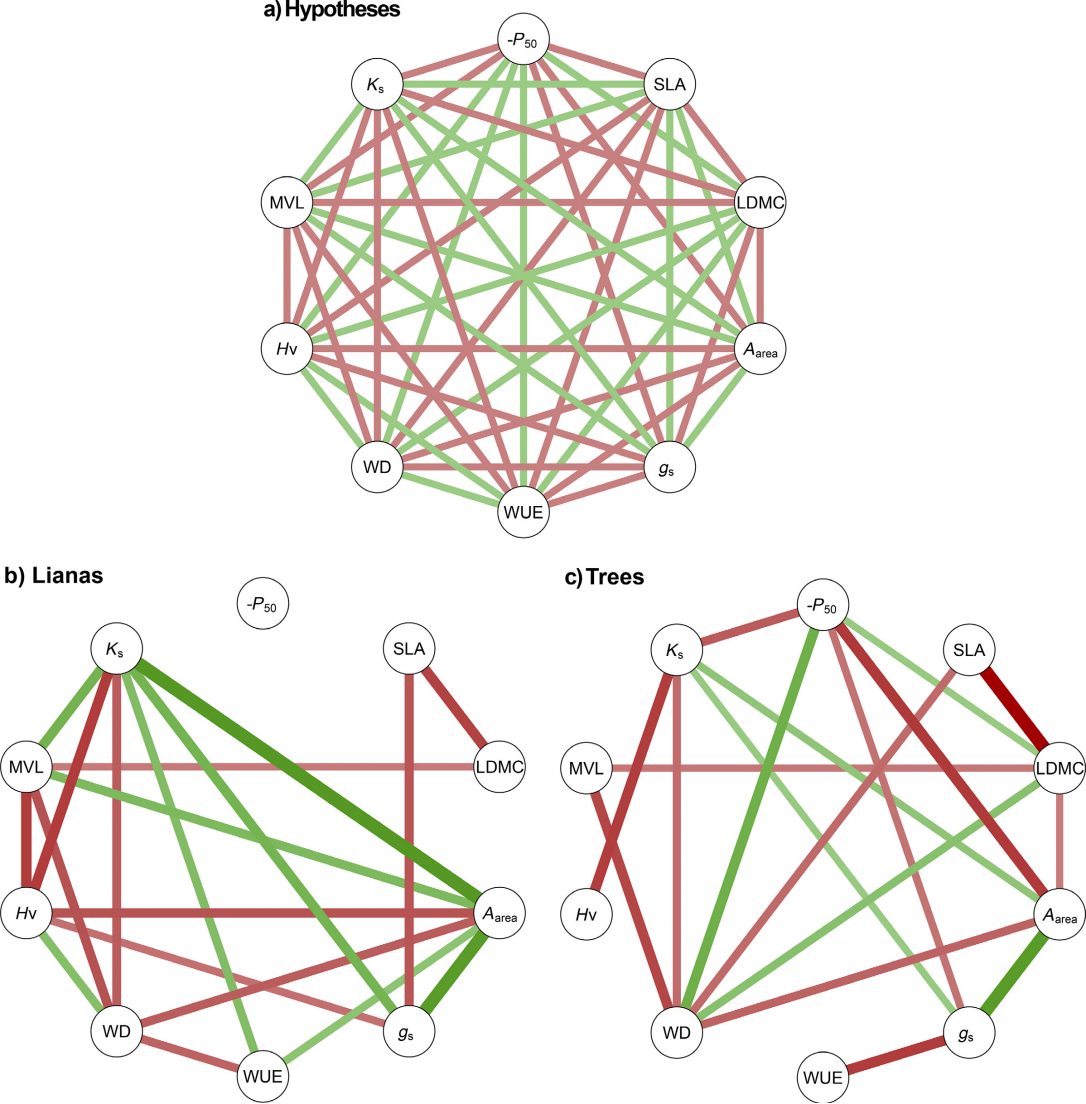
(a) Hypothesized relationships and results for (b) lianas and (c) trees between hydraulic safety (−*P*_50_, i.e., *P*_50_ multiplied by −1), hydraulic efficiency (*K*_s_) and other stem traits (left half of each panel) and leaf traits (right half of each panel). Other traits are specific leaf area (SLA), leaf dry matter content (LDMC), photosynthetic efficiency (*A*_area_), stomatal conductance (*g*_s_), water use efficiency (WUE), wood density (WD), Huber value (Hv), and maximum vessel length (MVL). Green lines indicate positive correlations and red lines indicate negative correlations, and the width in panels b and c corresponds to the correlation strength. The hypothesized relationships are similar for lianas and trees. Nonsignificant correlations in panels b and c are not shown. For correlation coefficients, see Appendix S11.

Here, we evaluate the associations among hydraulic efficiency, hydraulic safety (here measured as cavitation resistance, which is the xylem potential at 50% loss of hydraulic conductivity; *P*_50_), a suite of relevant physiological and morphological traits (wood density, maximum vessel length, Huber value, water use efficiency, specific leaf area, leaf dry matter content, leaf photosynthetic efficiency, and stomatal conductance), and abundance for saplings of 24 liana and 27 tree species from two tropical moist forests in Central Panama. We ask three questions. First, do lianas and trees differ in the trade‐off between hydraulic efficiency and hydraulic safety? We expected that, although lianas and trees may differ in their average trait values because of less investment in supporting tissue for lianas, the classical trade‐off between hydraulic efficiency and safety would be similar between lianas and trees, as traits that promote efficiency (e.g., wide and long vessels) should reduce safety regardless of life form. Second, do lianas and trees differ in associations of hydraulic efficiency or safety with other physiological and morphological traits? We expected hydraulic efficiency to be positively related to maximum vessel length, photosynthetic efficiency, stomatal conductance and specific leaf area, and negatively to leaf dry matter content and wood density, water use efficiency, and Huber value (sapwood area/leaf area) (Fig. 1a). Based on the trade‐off between efficiency and safety, we expected hydraulic safety to be oppositely related to these variables. We expected that both hydraulic efficiency and safety are “anchor” traits, meaning that they are strongly correlated to other traits because of their importance for plant functioning. Third, how are these traits related to the abundance of liana and tree saplings in wet tropical forests? We expected that shade‐tolerant species of lianas and trees with conservative trait values (e.g., high wood density and low specific leaf area) would be more abundant in these relatively dense and wet forests. Hydraulic safety would be less important because species are rarely water limited and experience low hydraulic risk, and hydraulic efficiency would be less important because of low light conditions and low transpiration rates in the understory.

## Materials and Methods

### Forest sites

We collected data from saplings of liana and tree species in two forest sites in Central Panama: San Lorenzo and Soberania national parks. We included these two sites to expand the range in life‐history strategies among our focal species and test for the generality of the results (Condit et al. 2000, Engelbrecht et al. 2007). In San Lorenzo, data were collected along the road leading to the Smithsonian Tropical Research Institute's Canopy Crane (9°16′51.71″ N, 79°58′28.27″ W), and in Soberania along Pipeline road (9°8′11.54″ N, 79°43′24.71″ W), near the Gamboa Research facilities. We collected individuals along roads growing under similar light levels. Both forests have a distinct dry season: San Lorenzo national park is located on the Caribbean coast and receives 3,203 mm rainfall per year at the canopy crane site (140 m above sea level), with a 3‐month dry season (<100 mm rainfall per month) from January until March. Pipeline road, which crosses the Soberania national park, is located near the center of the Isthmus of Panama and the area where we undertook our sampling (70 m above sea level) receives ~2,311 mm rainfall per year, with a 4‐month dry season (<100 mm rainfall per month) from mid‐December until mid‐April. Mean annual and monthly rainfall was calculated from 20‐ to 80‐yr rainfall records in a network of rainfall stations (S. Paton, *personal communication*). We will refer to the Soberania forest as the moist forest and to San Lorenzo as the wet forest because of their differences in rainfall. Both sites have clayey soils with a pH around 5 (Turner and Engelbrecht 2011) and a mean annual temperature of 26°C. Due to rapid species turnover across the rainfall gradient (Condit 1998), focal species largely differed among sites.

### Species selection

In this study, we analyzed plant hydraulic traits for liana and tree species. We selected these species based on variation in life history strategies (mainly for trees, data for lianas are rare), and variation in growth form for lianas. A total of 13 tree and 13 liana species were collected from San Lorenzo national park and 16 tree and 13 liana species were collected from Soberania national park. From the 10 liana species from Soberania national park for which we had growth form information, one‐half was self‐supporting and one‐half was structural parasites in the sapling stage (see van der Sande et al. 2013). For the other species, we lacked this information. Results on differences in traits between lianas and trees based on the species from Soberania national park were published earlier (van der Sande et al. 2013). Two tree and two liana species were collected in both forest sites, and all other species were unique to the sites. Hence, in total, we collected data for 51 species: 27 tree and 24 liana species. We focused on saplings between 1.5 and 2.0 m tall because of the importance of the regeneration stage for species adaptations (Poorter 2007), their limited root system may cause potentially stronger water limitation and, hence, increase the importance of hydraulic traits. Moreover, destructive sampling was not feasible for adult individuals, and seedlings often have undifferentiated xylem conduits that are impossible to measure. We selected five individuals per species per site. Collected saplings of lianas and trees were growing in similar intermediate to high light environments along unpaved forest roads, in order to minimize potential phenotypic trait variation caused by differences in environmental conditions. Field sampling took place between February and July 2011 (i.e., in the dry season).

### Hydraulic efficiency and safety

For three to four randomly selected individuals per species, we measured maximum xylem‐specific conductivity (i.e., hydraulic efficiency) after removing xylem embolisms, and xylem cavitation resistance (i.e., hydraulic safety) using the pressure sleeve method (Cochard et al. 1992, Salleo et al. 1992). The aboveground shoots of saplings were harvested from the field between 08:00 and 10:00, stored in a large, dark cooler to avoid excessive dehydration and transported to a laboratory for further processing. Lateral branches and leaves were cut from the main stem, and cuts were sealed with instant Loctite super glue. Stems were re‐cut under distilled water, and distal ends were trimmed with a razorblade to clear any blocked vessels. The stems were cut to a length 10% longer than the maximum vessel length (MVL; see Trait measurements) to avoid open vessels that can affect measurements of hydraulic conductivity (on average the stems were 84 cm long). We took caution to avoid open vessels, because these can lead to overestimation of the vulnerability curves when using the pressure sleeve method (Martin‐StPaul et al. 2014). The bark was removed from about 1 cm of the shoot ends. While submerged, the shaved basal end of the stem was wrapped in Parafilm (Pechiney Plastic Packaging, Chicago, Illinois, USA) and connected to a manifold of hysteresis‐resistant polytetrafluoroethylene (PTFE) tubing, holding up to five stems at a time. With all stems in place, the manifold was attached to a Scholander pressure chamber (Model 600; PMS Instruments, Albany, New York, USA) at 150 kPa overhead pressure filled with a flow solution of 10 mmol potassium chloride (KCl) in distilled, degassed, and filtered (0.2 μm) water. Stems were flushed for at least 30 min to remove xylem emboli, after which the manifold was attached to an overhead water reservoir (Sperry et al. 1988), supplying the same flow solution to the stems at 5 kPa for 10 min before measuring conductivity. Three repeated measurements were taken to assure that conductivity had reached a steady flow and measured as the time needed to fill 1 mL of a fine grated serological pipette. Solution injection always followed the direction of natural water flow in the plant, from the base to the top.

Subsequently, we determined cavitation resistance by applying increasing air pressure (i.e., to mimic tissue desiccation) using a pressure sleeve (PMS Instruments, Albany, New York, USA; Cochard et al. 1992, Salleo et al. 1992) to the stem and measuring the conductivity. After pressurizing, stems were left to rest for 10 min with both ends under water, after which they were reconnected to the low‐pressure head flow system and conductance was measured. This sequence was repeated with increasing sleeve pressures, using steps of 0.5 MPa if conductivity declined fast, and steps of 1 or 2 MPa if conductivity declined slowly (see Appendix S1–S4), until conductance had declined by more than 90%. From these measurements, we constructed one sigmoidal vulnerability curve per species (Cochard et al. 2013), with loss in hydraulic conductivity as a function of xylem water potential (see Appendices S1–S4). We constructed these curves using Nonlinear Least Squares regression analyses using the nls function in R. Loss in hydraulic conductivity (in percent) was calculated as follows: 100 – (*K*_*x*_/K_max_ × 100), in which *K*_*x*_ is the conductance (mol·s^−1^·MPa^−1^) measured after pressurizing and *K*_max_ the maximum hydraulic conductance measured after flushing. From the vulnerability curves, we calculated the xylem potential at 50% loss of hydraulic conductivity (*P*_50_). *P*_50_ is the most commonly used measure to characterize cavitation resistance or xylem safety. We also calculated the xylem potential at 12% and 88% loss of hydraulic conductivity (i.e., *P*_12_ and *P*_88_, respectively), because these are sometimes used as alternative measures of xylem safety (Domec and Gartner 2001, Choat et al. 2012, Gleason et al. 2015). *P*_50_ (and *P*_12_ and *P*_88_) values were multiplied by −1 so that high values indicate high cavitation resistance and low values indicate low cavitation resistance. Maximum sapwood‐specific hydraulic conductivity (*K*_s_; mol·m^−1^·s^−1^·MPa^−1^) was calculated by dividing maximum hydraulic conductivity (*K*_max_; in mol·s^−1^·MPa^−1^·*m* ^−1^, where *m* refers to the length of the stem in meters) by the sapwood area in m^2^ (see Trait measurements), and the maximum leaf‐specific hydraulic conductivity (*K*_l_) by dividing *K*_max_ by the total leaf area above the apical cut in m^2^. Results of *K*_s_ and *K*_l_ were qualitatively similar, and therefore, results of *K*_s_ will be presented in the manuscript and of *K*_l_ in an appendix. *K*_s_ indicates the optimization of wood hydraulic function per xylem volume and does not necessarily affect total water transport. Sapwood area was estimated after removing the bark and subtracting the pith and measured with a caliper. All transversal wood area was considered to be functional because of the young age of the plants. We will refer to *P*_50_ multiplied by −1 (i.e., resistance to cavitation) as “hydraulic safety” and to the maximum sapwood‐specific hydraulic conductivity as “hydraulic efficiency.”

### Trait measurements

For all five individuals per species, additional morphological and physiological whole plant, stem, and leaf traits were collected; wood density (WD; g/cm^3^), maximum vessel length (MVL; cm), Huber value (Hv; sapwood area/leaf area; cm^2^/cm^2^), specific leaf area (SLA; cm^2^/g), leaf dry matter content (LDMC; g/g), leaf area‐specific photosynthetic efficiency (*A*_area_; μmol·m^−2^·s^−1^), stomatal conductance (*g*_s_; mmol·m^−2^·s^−1^), and water use efficiency calculated as *A*_area_/*g*_s_ (WUE; μmol/mol). These traits were included because they represent the leaf, stem, and whole plant economic spectra (Wright et al. 2004, Chave et al. 2009, Díaz et al. 2015), are important for species’ growth rate and ecological strategy (Poorter et al. 2008, Wright et al. 2010) and often correlated with hydraulic traits in trees (Santiago et al. 2004, Markesteijn et al. 2011a,b). High Huber values are associated with a conservative strategy because low leaf area reduces transpiration, and narrow xylem vessels that reduce cavitation risk have relatively more vessel wall tissue, which increases sapwood area.

Leaf traits were determined on a pooled sample of five leaves per individual, and stem traits were determined based on one stem sample per individual, excluding the bark. WD, SLA, and LDMC were measured according to general protocols (Perez‐Harguindeguy et al. 2013). The Hv was calculated as the sapwood area at the upper distal cut divided by total leaf area it supported. MVL was measured with the air injection method (Greenidge 1952); we pressurized the stems at 1–1.5 bar and re‐cut them under water, about 1 cm at a time, until air bubbles emerged, indicating that the longest vessel element had been opened and found. We used MVL as a proxy for mean vessel length, as the two are strongly correlated (Jacobsen et al. 2012), and because of its relation with other important life history traits (Markesteijn et al. 2011b). Physiological traits (*A*_area_, *g*_s_, and WUE) were determined on five different individuals per species in the field at the start of the wet season. Maximum photosynthesis per unit leaf area (*A*_area_) and stomatal conductance (*g*_s_) were measured between 07:00 and 11:00 using a LI‐COR 6400xt (Li‐Cor, Lincoln, Nebraska, USA) at an irradiance of 1,000 μmol·s^−1^·m^−2^. For more details about collection and measurements of these morphological and physiological traits, see Van der Sande et al. (2013).

### Species’ abundance data

To obtain the abundance of the liana and tree species, we used four existing 1‐ha forest plots (two moist forest plots and two wet forest plots) adjacent to the respective areas where we sampled the liana and tree hydraulics and traits. These four 1‐ha plots are part of a larger set of eight plots that span the rainfall gradient across the Panamanian isthmus and that were established in 2013 with grant funding from the UK Natural Environment Research Council (NERC) to Owen Lewis and faculty startup funds to Liza Comita. In each of the 1‐ha forest plots, established seedlings and saplings (≥20 cm tall and < 1 cm dbh) were censused and measured multiple times between 2013 and 2017 in 400 seedling plots of 1 m^2^ placed at 5‐m intervals in each 1 ha plot (N = 1600). Data from the last census of the seedlings and saplings in January 2017, funded by a U.S. National Science Foundation (NSF) RAPID grant, were used to estimate the abundance for our selected species in the respective forest types. The data from the two moist forest plots and the two wet forest plots were pooled for analyses, to obtain a more accurate estimate of abundance based on a larger sample size.

### Analyses

Trait differences between lianas and trees have been evaluated for part of the data set in a previous paper (van der Sande et al. 2013) and are summarized for the entire data set in Appendix S5. Here, we evaluate how lianas and trees differ in the relationship between hydraulic efficiency (maximum sapwood‐specific conductivity; *K*_s_) and hydraulic safety (*P*_50_ multiplied by −1), by testing for differences in slope between the two life forms using standardized major axis (SMA) analysis. Standardized major axis analyses can test bivariate relationships (hence, without clear cause‐and‐effect variables) and differences in slope among groups. We used species average values for all traits. Initially, forest type was included as a factor in the analyses, but since forests showed no significant differences (Appendix [Link], [Link]), and since including random factors in SMA analysis is not possible, we simplified the model and combined species from both forest types. For the species that were measured in both forest types, we used average trait values to avoid pseudo‐replication. To evaluate whether there is an upper ceiling relationship between efficiency and safety, we also evaluated differences between lianas and trees in the 90% quantile relationship between hydraulic efficiency and hydraulic safety using quantile regression analysis. Possible phylogenetic signals in hydraulic safety and efficiency were tested with Pagel's lambda, which generally performs well for testing phylogenetic signal in complex systems (ucodep>n/ucodep>nkemüller et al. 2012), using the phylosig function of the phytools package in R (Revell 2012).

To evaluate how lianas and trees differed in their associations of hydraulic efficiency and hydraulic safety with morphological and physiological traits, we used similar SMA analyses. Again, traits of species that were measured in both forest types were averaged, because very few trait associations differed between forest types (only between hydraulic efficiency and Hv for lianas, between hydraulic efficiency and SLA for trees, and between hydraulic safety and WD for trees; Appendices S6 and S7). In total, we performed 17 SMA analyses with a critical *P* level of 0.05. In order to correct for the probability to falsely reject the null hypotheses, we also calculated the Benjamini‐Hochberg‐corrected *P* values (Benjamini and Hochberg 1995). Furthermore, to explore associations among all traits for lianas and trees, we calculated pairwise Pearson correlations and presented these in a correlation network for lianas and trees separately and forest types combined, and performed principal component analyses for lianas and trees separately (after scaling the traits by dividing by their standard deviation).

The relationship between species abundance and traits was evaluated using generalized linear models with a negative binomial error distribution per trait. We used a negative binomial error distribution because this gave a better goodness‐of‐fit (using a chi‐square test) than a Poisson distribution (Appendix S8). In each model, an interaction between the trait and life form was included to evaluate differences in trait effects on abundance between lianas and trees.

Analyses were performed in R v. 3.3.1 (R Core Team 2017). SMA analyses were done with the sma function of the smatr package (Warton et al. 2012), Pearson correlations with the rcorr function of the Hmisc package (Harrell et al. 2018), principal component analyses using the rda function of the vegan package (Oksanen et al. 2018), and quantile regression analyses using the qr function of the quantreg package (Koenker 2018). For negative binomial generalized linear models, we used the nb.glm function of the MASS package (Venables and Ripley 2002), and for the Poisson generalized linear model, we used the glm function. Chi‐square tests to evaluate goodness‐of‐fit were performed using the pchisq function.

## Results

Lianas had higher hydraulic efficiency (i.e., sapwood‐specific maximum hydraulic conductivity; *K*_s_) and lower hydraulic safety (measured as cavitation resistance; −*P*_50_) than trees (Appendix S5), similar to what we found in an earlier study for only part of these data (van der Sande et al. 2013). Furthermore, lianas had lower Hv and higher *A*_area_ and *g*_s_ than trees, indicating that lianas have a more acquisitive growth strategy (Cai et al. 2009, Zhu and Cao 2009). None of the traits differed significantly between self‐supporting and structural parasite liana species (based on a comparison of only 10 species for which we had this information; Appendix S9), in line with our earlier findings (van der Sande et al. 2013).

**Figure 2:**
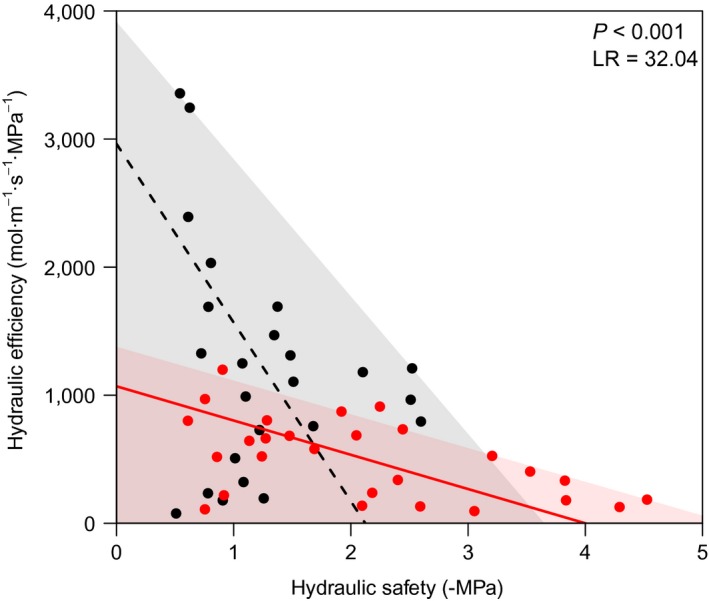
Relationship between hydraulic efficiency (i.e., maximum sapwood hydraulic conductivity) and hydraulic safety (i.e., water potential at 50% loss of hydraulic conductivity multiplied by −1) for lianas (black) and trees (red). The lines represent the estimated relationship between the two variables (Table 1), the solid line indicates a significant relationship (for trees) and dashed line indicates a nonsignificant trend (for lianas). The gray and red background represent the upper 90% quantiles (which were significant for both lianas and trees). The likelihood‐ratio test (LR) with corresponding significance (*P*) for the difference in slope between lianas and trees is given. For statistics of the individual slopes, see Table 1.

Lianas and trees differed in the trade‐off between hydraulic efficiency and hydraulic safety: trees showed a trade‐off (*P* = 0.006, *R* ^2^ = 0.27) but lianas did not (*P* = 0.234, *R* ^2^ = 0.06; Fig. 2, Table 1). Both life forms showed a significant negative upper ceiling (i.e., 90% quantile) relationship between hydraulic efficiency and hydraulic safety, indicating that high efficiency and high safety do not occur in combination (Fig. 2). This upper ceiling relationship, however, was stronger for trees than lianas. Results were similar for leaf hydraulic conductivity (*K*_l_) and when hydraulic safety was assessed using the water potential at 12% and 88% conductivity loss, which have been used as alternative safety measures (Domec and Gartner 2001, Gleason et al. 2015; Appendix S10). Hydraulic safety had a strong phylogenetic signal (Pagel's lambda = 0.97, *P* = 0.030) but hydraulic efficiency only a weak one (lambda = 0.25, *P* = 0.365).

**Table 1:** Results of standardized major axis analyses to test how lianas and trees differ in their relationships between hydraulic efficiency, safety, and other stem and leaf traits

Var 1	Var 2	Lianas			Trees			Differences in slope of lianas vs. trees		
		Slope	*P*	*r* ^2^	Slope	*P*	*r* ^2^	LR	*P*	BH‐corrected *P*
Hydraulic efficiency	Hydraulic safety	−1.98	0.234	0.06	−0.38	0.006	0.27	32.04	<0.001	<0.001
Hydraulic efficiency	WD	−1.09	0.001	0.43	−1.12	0.047	0.15	0.01	0.904	0.932
Hydraulic efficiency	MVL	1.06	0.001	0.42	1.08	0.211	0.06	0.01	0.932	0.932
Hydraulic efficiency	Hv	−0.72	<0.001	0.62	−1.39	<0.001	0.42	9.61	0.002	0.009
Hydraulic efficiency	WUE	0.66	0.007	0.29	−2.70	0.509	0.02	24.27	<0.001	<0.001
Hydraulic efficiency	SLA	−0.74	0.127	0.10	−2.59	0.741	<0.01	17.75	<0.001	<0.001
Hydraulic efficiency	LDMC	1.16	0.811	<0.01	1.06	0.981	<0.01	0.10	0.758	0.859
Hydraulic efficiency	*A*_area_	0.81	<0.001	0.75	1.05	0.066	0.13	1.45	0.229	0.354
Hydraulic efficiency	*g*_s_	0.89	<0.001	0.57	0.99	0.064	0.13	0.18	0.670	0.814
Hydraulic safety	WD	1.82	0.619	0.01	0.82	0.001	0.37	8.56	0.003	0.010
Hydraulic safety	MVL	−1.77	0.409	0.03	−0.79	0.059	0.13	7.85	0.005	0.014
Hydraulic safety	Hv	−1.20	0.291	0.05	1.01	0.484	0.02	0.36	0.546	0.714
Hydraulic safety	WUE	−1.31	0.352	0.04	1.02	0.844	<0.01	0.70	0.404	0.572
Hydraulic safety	SLA	−1.47	0.082	0.13	−0.98	0.255	0.05	2.00	0.158	0.269
Hydraulic safety	LDMC	1.77	0.054	0.16	0.84	0.036	0.16	7.18	0.007	0.017
Hydraulic safety	*A*_area_	−1.30	0.343	0.04	−0.66	0.001	0.37	6.39	0.011	0.021
Hydraulic safety	*g*_s_	−1.49	0.925	0.00	−0.72	0.021	0.19	6.51	0.011	0.021

*Notes*:

Each row represents one model in which the slope of lianas, trees, and their difference are tested. The slope, *P*, and *r* ^2^ are given per life form, as well as the likelihood‐ratio (LR) test, *P* value and Benjamini‐Hochberg (BH)‐corrected *P* values for the difference in slope between lianas and trees. We used the BH‐corrected *P* values because this is a powerful tool to correct for the probability to wrongly reject the null hypotheses with multiple comparisons (Benjamini and Hochberg 1995). To facilitate comparison among models, the value of the slope is based on the scaled variables, that is, by subtracting the mean and dividing by the standard deviation. −*P*_50_, hydraulic safety; *K*_s_, hydraulic efficiency; SLA, specific leaf area; LDMC, leaf dry matter content; *A*_area_, photosynthetic efficiency; *g*_s_, stomatal conductance; WUE, water use efficiency; WD, wood density; Hv, Huber value; and MVL, maximum vessel length.

**Figure 3:**
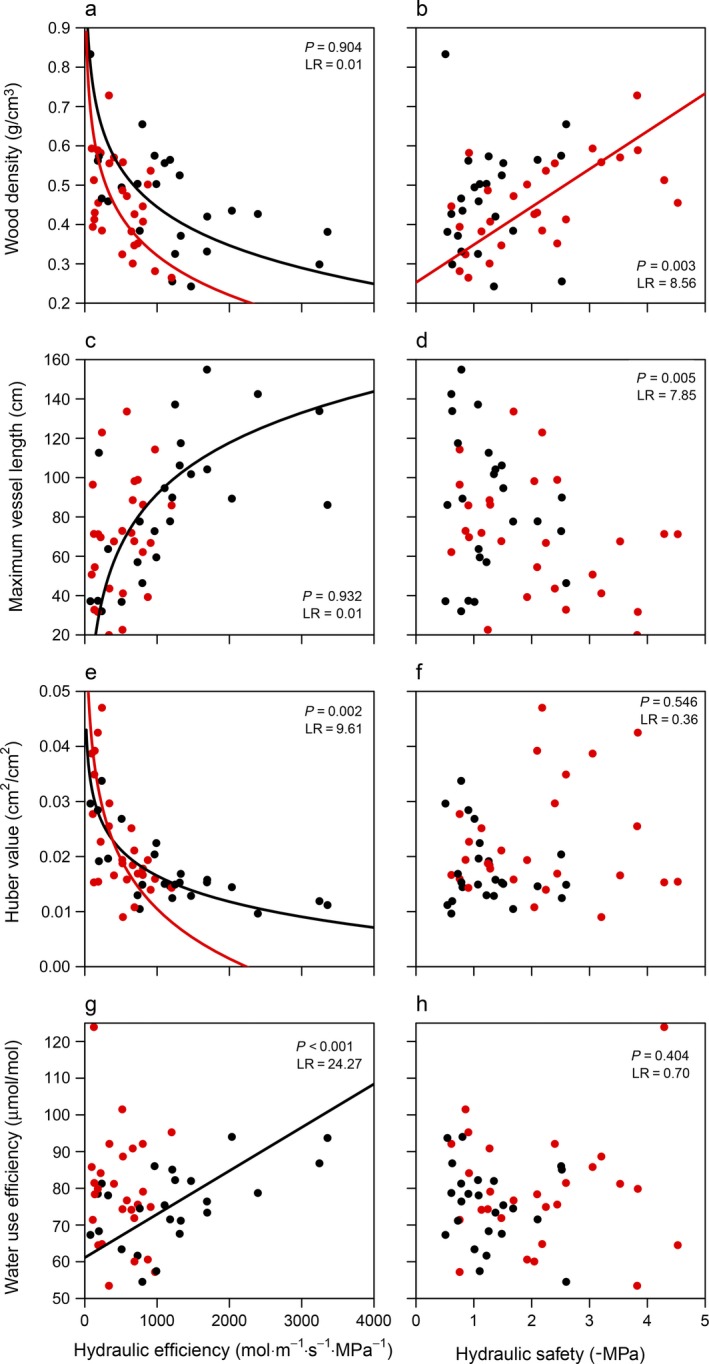
Relationships of hydraulic efficiency (left column) and hydraulic safety (right column) with morphological and physiological traits; wood density (a, b), maximum vessel length (c, d), Huber value (e, f; sapwood area/leaf area), and water use efficiency (g, h). The relationships are tested using standardized major axis regressions (Table 1). Data for lianas are given in black; data for trees are given in red. Trend lines for non‐significant relationships (*P* > 0.05) are not shown. The likelihood‐ratio test (LR) with corresponding significance (*P*) for the difference in slope between lianas and trees is given. For statistics of the individual slopes, see Table 1.

**Figure 4:**
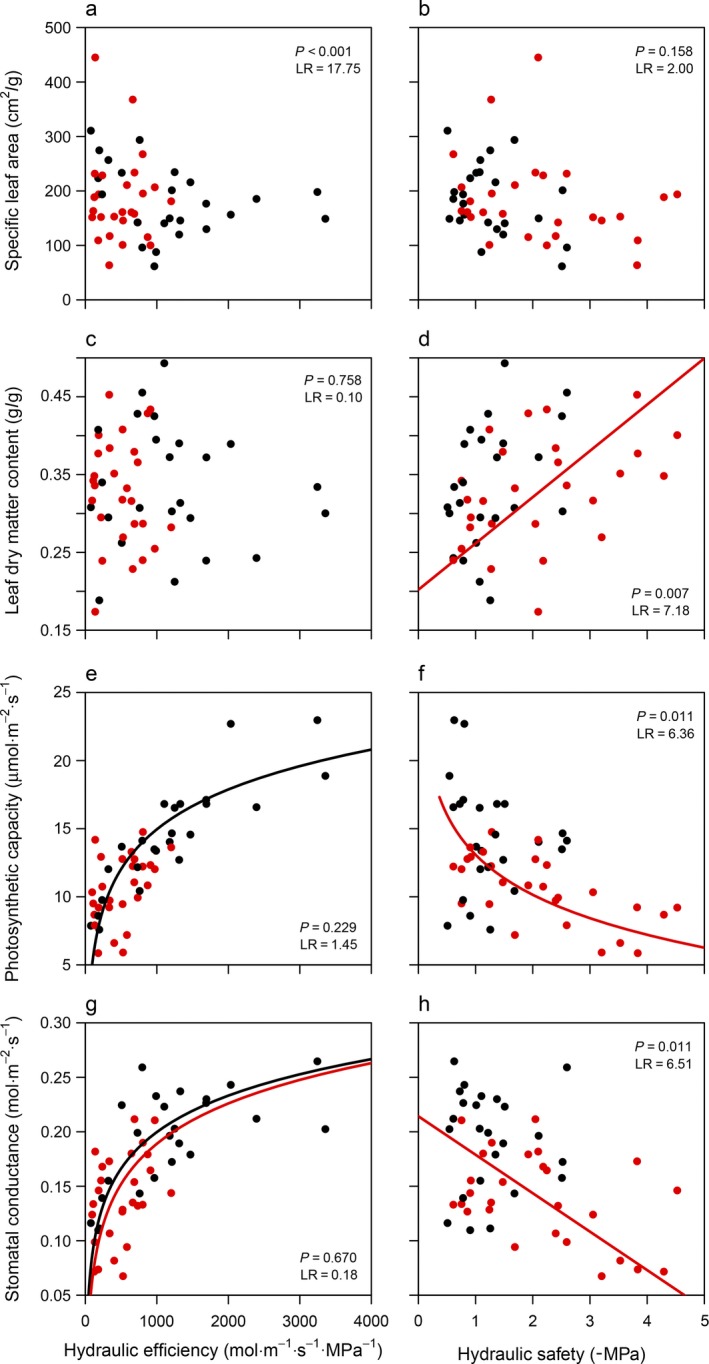
Relationships of hydraulic efficiency (left column) and hydraulic safety (right column) with morphological and physiological leaf traits; (a, b) specific leaf area, (c, d) leaf dry matter content, (e, f) area‐based photosynthetic efficiency, and (g, h) stomatal conductance, tested using standardized major axis analyses (Table 1). Data for lianas are given in black; data for trees are given in red. Trend lines for nonsignificant relationships (*P* > 0.05) are not shown. The likelihood‐ratio test (LR) with corresponding significance (*P*) for the difference in slope between lianas and trees is given. For statistics of the individual slopes, see Table 1.

**Figure 5:**
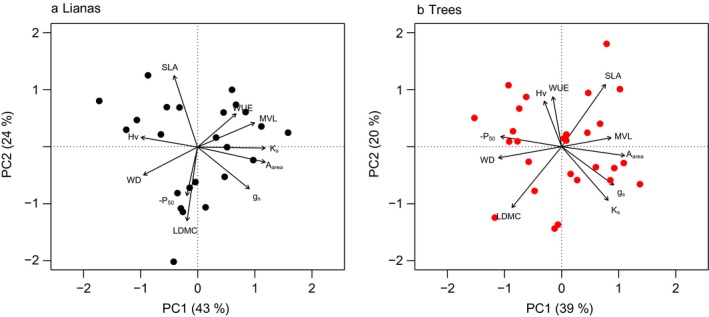
Principal component analyses for (a) lianas and (b) trees based on the two forest types combined. Each point represents one species. The traits included are as follows: hydraulic efficiency (*K*_s_), hydraulic safety (−*P*_50_), wood density (WD), maximum vessel length (MVL), Huber value (Hv), water use efficiency (WUE), specific leaf area (SLA), leaf dry matter content (LDMC), area‐specific photosynthetic efficiency (*A*_area_), and stomatal conductance (*g*_s_).

Standardized major axis analyses (Fig. 3 and 4), correlation networks (Fig. 1b, c; Appendix S11) and principal component analyses (Fig. 5) showed that hydraulic efficiency was similarly correlated for lianas and trees to most other traits; hydraulic efficiency was positively correlated with MVL, *A*_area_, *g*_s_ (though not significantly for trees for MVL and *g*_s_), and negatively with WD and Hv (*R* ^2^ ranging between 0.13 and 0.42). However, the correlation between hydraulic efficiency and Hv was more negative for trees (standardized slope = −1.39; Table 1, Fig. 3e) than for lianas (standardized slope = −0.72), and the correlation between hydraulic efficiency and WUE was positive for lianas (*R* ^2^ = 0.29) but nonsignificant for trees (*R* ^2^ = 0.02; Table 1, Fig. 3g). The correlation between hydraulic safety and other traits, however, differed largely between lianas and trees (Fig. 3, 4, Table 1); hydraulic safety of lianas was not correlated with any of the morphological and photosynthetic traits, whereas hydraulic safety of trees was positively correlated with WD and LDMC and negatively with *A*_area_ and *g*_s_ (*R* ^2^ ranging between 0.16 and 0.37). Hence, hydraulic efficiency is strongly correlated with a suite of traits especially in lianas, whereas safety is strongly correlated only with a suite of traits in trees. See Appendix S13 for within‐species correlation analyses.

WD was strongly correlated with many traits for both life forms, but SLA only weakly (Fig. 1b, c), indicating that SLA, as the main component of the leaf economics spectrum, poorly correlates with hydraulic and morphological traits. Stomatal conductance (*g*_s_) was negatively associated with water use efficiency (WUE) for trees, because high conductance leads to high water loss and thus low WUE. However, there was no correlation between *g*_s_ and WUE for lianas, and a weak positive correlation between *A*_area_ and WUE. This means that the WUE of lianas is mainly driven by variation in *A*_area_, whereas WUE of trees is mainly driven by variation in *g*_s_.

**Figure 6:**
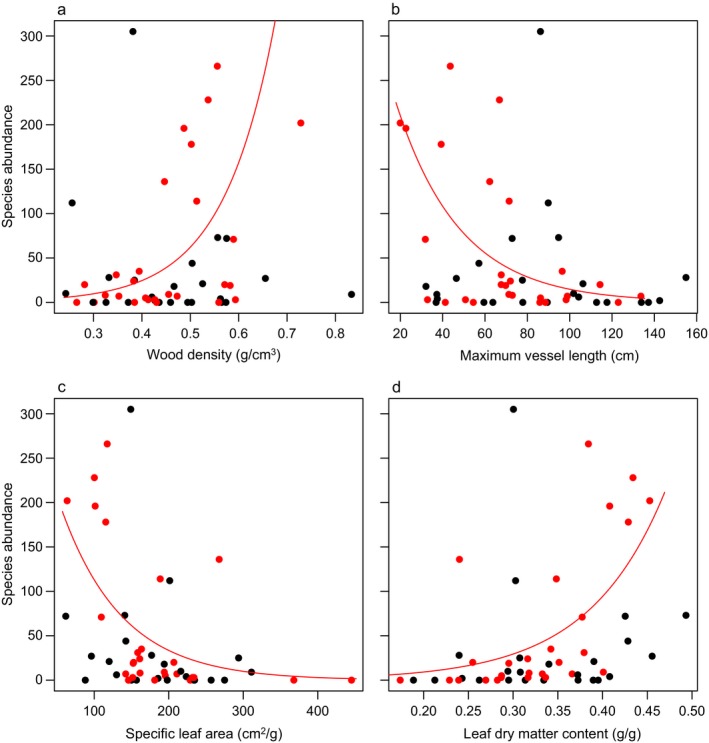
Relationship of a) wood density, b) maximum vessel length, c) specific leaf area and d) leaf dry matter content with species abundance across 800 m^2^. Species abundance data came from 1‐ha plots adjacent to the sites where traits were measured. Within each plot, sapling abundance was measured in 400 1‐m^2^ subplots, totaling a measured area of 800 m^2^ per species. Red lines indicate significant relationships for trees. None of the relationships was significant for lianas. For statistics, see Appendix S14.

Hydraulic efficiency and safety did not affect abundance of tree and liana species (Fig. 6; Appendix S14). Abundance of tree saplings was positively related to WD and LDMC and negatively to SLA and MVL, but abundance of liana saplings was not related to any of the measured traits (Appendix S14).

## Discussion

We evaluated how lianas and trees differ in the associations between hydraulic efficiency, hydraulic safety, and other physiological and morphological traits, and the influence of traits on local abundance. We showed that, while hydraulic efficiency and safety showed a trade‐off in trees, they were decoupled in lianas. None of the liana or tree species had both high efficiency and high safety. Lianas and trees also showed pronounced differences in trait coordination. Conservative traits increased abundance in trees but not in lianas, and hydraulic efficiency and safety did not affect species abundance. These results indicate that different mechanisms determine hydraulic efficiency, safety and abundance in lianas and trees.

### The hydraulic efficiency–safety trade‐off for lianas and trees

We expected that hydraulic efficiency (maximum sapwood‐specific conductivity; *K*_s_) and hydraulic safety (the xylem potential at 50% loss of hydraulic conductivity multiplied by −1; −*P*_50_) would be negatively correlated because xylem traits that enhance hydraulic efficiency come at the expense of safety and vice versa (Lens et al. 2011). For example, long and wide vessels increase hydraulic conductivity (Lens et al. 2011), but simultaneously increase the risk of cavitation because wide vessels have a higher probability of containing a large pit membrane pore (Wheeler et al. 2005). For trees, we indeed found a strong trade‐off between efficiency and safety, but not for lianas (Fig. 2), indicating that lianas can avoid the efficiency–safety trade‐off. Nevertheless, both lianas and trees showed an upper limit relation between efficiency and safety (Fig. 2) and, while some liana species reached very high efficiencies, their safety was generally low. These results show that it is impossible to combine high hydraulic efficiency and high safety (Gleason et al. 2015, Bittencourt et al. 2016). This upper limit may be caused by wide vessels that enhance hydraulic efficiency and are likely to contain large pits that lead to high cavitation risk, and/or by the trade‐off between the investment in conduits to enhance hydraulic efficiency at higher water potentials vs. the investment in fiber to prevent implosion at very negative water potentials (Bittencourt et al. 2016). The species in our study (both lianas and trees) showed about one‐half the range in hydraulic safety as is found globally (Choat et al. 2012), probably because greater hydraulic safety is unnecessary in these wet forests. In a slightly drier forest (1,865 mm/yr) and for more exposed adult canopy individuals, lianas and trees combined showed a trade‐off between efficiency and safety (De Guzman et al. 2016), perhaps because these species function closer to their upper efficiency and safety limits. Across all our liana and tree species, we also found a significant negative correlation between hydraulic efficiency and safety (*r* = −0.42, *P* = 0.002, df = 49), indicating that collectively evaluating life forms can conceal group differences.

Even though lianas have generally lower hydraulic safety than trees (Fig. 2; Appendix S5), they may not experience higher levels of cavitation because of (1) access to deeper water sources (Andrade et al. 2005, Schnitzer 2005, Chen et al. 2015), (2) stronger stomatal control to avoid excessive water loss (Chen et al. 2015), and (3) stronger leaf osmotic adjustment to maintain cell turgor at lower leaf water potentials (Maréchaux et al. 2017). These alternative or complementary ways to avoid desiccation under drought conditions may allow the coexistence of liana species with low safety and liana and tree species with much higher safety (Fig. 2). Strategies of low safety and high efficiency, however, may lead to higher mortality at extreme levels of drought (Nepstad et al. 2007). Surprisingly, several species, both lianas and trees, had low efficiency and low safety, a combination that we would expect to be outcompeted or remain very rare in both wet and dry forest ecosystems. Hydraulic safety had a strong phylogenetic signal but hydraulic efficiency did not, indicating that the existence of species with low efficiency and low safety is not because of phylogenetic constrains. Globally, a large part of the woody species has low efficiency and low safety (Gleason et al. 2015). Possibly, the drought tolerance of species with low efficiency and low safety is determined by drought‐avoiding traits such as rooting depth and stomatal control, or by different wood volumes.

### What explains the uncoupling between hydraulic efficiency and safety for lianas?

Lianas may have higher hydraulic efficiency than trees (Zhu and Cao 2009, van der Sande et al. 2013, De Guzman et al. 2016; Appendix S5) because of their lower investment in supporting tissues such as fibers (Ewers et al. 2015), allowing for more investment in conducting tissue. It could therefore be that lianas have higher sapwood‐specific conductivity and are less constrained in the allocation of xylem to supporting and conducting tissue, and that more variation in hydraulic efficiency is possible at low safety (Bittencourt et al. 2016). The decoupling between hydraulic efficiency and safety may be explained by properties that affect only efficiency or only safety. For example, inter‐vessel pit membrane properties can vary independently from vessel size or length (Hacke and Sperry 2001) and can affect hydraulic safety more than efficiency (Tyree and Sperry 1989, Maherali et al. 2006, Venturas et al. 2017). Pit pores limit the spread of air much more strongly than they limit the spread of water between adjacent vessels and are therefore thought to be especially important for controlling cavitation resistance (Wheeler et al. 2005, Hacke et al. 2006, Choat et al. 2007) while having less influence on water transport efficiency. Furthermore, high calcium concentrations in the pit membranes decrease flexibility of the membranes, which especially limits spread of air (Herbette and Cochard 2010). Therefore, if pit membrane properties differ between lianas and trees, then this could potentially explain why hydraulic efficiency and safety are decoupled in lianas but not in trees. Further studies are needed to evaluate the mechanisms underlying the uncoupling of safety and efficiency in lianas, from cellular to whole‐plant level, and why trees are not similarly able to decouple safety and efficiency.

### Efficiency as an anchor trait for lianas and efficiency and safety as anchor traits for trees

We expected that lianas and trees would have similar associations of hydraulic efficiency and safety with other traits; hydraulic efficiency would be positively associated with transporting tissues (maximum vessel length [MVL]), rates of gas exchange (i.e., physiologically active leaves with high specific leaf area [SLA], stomatal conductance [*g*_s_], and photosynthetic efficiency [*A*_area_]), and negatively with Huber value (Hv) and conservative traits that reduce water transport (high wood density [WD], leaf dry matter content [LDMC] and water use efficiency [WUE]). The relationships would be opposite for hydraulic safety. We found that hydraulic efficiency is correlated with many other traits for lianas and slightly more weakly for trees (Figs. 3, 4) and associated especially positively with gas exchange rates and negatively with Hv for lianas and trees (Fig. 5a, b). Hydraulic safety, however, was correlated with many other traits for trees, but was not correlated with any trait for lianas (Fig. 3, 4), and was associated most strongly positively with LDMC and negatively with SLA for lianas (Fig. 5a), and positively with WD and negatively with *A*_area_, *g*_s_, and MVL for trees (Fig. 5b). For lianas, the average correlation strength of hydraulic efficiency with other traits was higher (*r* = 0.55) than the average correlation strength of other traits (Appendix S15). For trees, both hydraulic efficiency (*r* = 0.30) and safety (*r* = 0.34) were among the traits most strongly correlated with other traits, together with *A*_area_ (0.35), *g*_s_ (0.32), and WD (0.37). Hence, hydraulic efficiency is an anchor trait associated with many other traits for lianas, while both hydraulic efficiency and hydraulic safety are anchor traits for trees.

The weak correlations of hydraulic safety of lianas with other traits may also indicate that different traits not studied here determine the safety of lianas (e.g., pit pore distribution, calcium control of membrane flexibility, or stomatal control). Hydraulic efficiency of lianas, however, is positively correlated with long vessels, low wood density, and high gas exchange rates. Contrary to expectations, efficiency of lianas is positively correlated with WUE, probably because WUE is more strongly driven by *A*_area_ than by *g*_s_ (Fig. 1b). This indicates that WUE of lianas is mainly determined by variation in carbon gain, which increases with hydraulic efficiency (Fig. 4e), and less by variation in water loss.

### Why is safety correlated with other traits for trees but not for lianas?

In these relatively wet tropical forests, hydraulic safety is strongly associated with other traits for trees. The positive correlations of safety with WD and LDMC, and negative correlations with *A*_area_ and *g*_s_, could indicate that tough wood and leaves with low physiological activity are an important strategy to enhance hydraulic safety. Possibly, low light availability in wetter forests increases the importance of conservative trait values, including high safety (Markesteijn et al. 2011a), and therefore results in strong trade‐offs of hydraulic safety and WD with other traits. Alternatively, the lack of correlations between hydraulic safety and other traits for lianas is caused by the small range in safety values among liana species (Fig. 2). When evaluating the relations of safety with WD, LDMC, *A*_area_, and *g*_s_ (which were significant for trees; Figs. 3b and 4d, f, h) using a similar range in safety values for trees as for lianas (necessarily also reducing the sample size from 26 to 20 tree species), none of the relationships are significant for trees (Appendix S12). This indicates that relationships of safety with other traits for lianas could be significant if a larger range in safety and/or a larger sample size is used, although other studies also report low safety values for lianas (De Guzman et al. 2016) and, hence, lianas with comparably high safety probably do not exist. However, the small range in efficiency values among tree species does still result in significant correlations between efficiency and other traits, suggesting that also for lianas the small range in safety values cannot fully explain the lack of correlations. The importance of hydraulic safety for trees and of hydraulic efficiency for lianas, in combination with the lack of high efficiency in trees and the lack of high safety in lianas, suggests that trees can tolerate dry and/or shady conditions, whereas lianas can avoid experiencing dry conditions (Schnitzer 2005).

### Conservative traits affect tree but not liana abundance in moist Panamanian forests

We expected that, in these wet but light‐limited forests, high hydraulic efficiency would not increase abundance because it provides no advantage under low‐light conditions, and high hydraulic safety would not increase abundance because species are rarely water limited in these wet forests. However, under the low light conditions species with conservative trait values such as low SLA, *A*_area_, and high WD would reach higher abundances at the sapling stage. We indeed found that hydraulic efficiency and safety did not affect species abundance of tree or liana saplings and that acquisitive traits (SLA, MVL) decreased and conservative traits (WD, LDMC) increased abundance of tree saplings (Fig. 6; Appendix S14). Strong light limitation in the understory of these moist and wet forests may provide an advantage to species with a conservative resource strategy, which survive best as saplings and therefore reach highest abundances. Moreover, conservative species may grow slowly into adult trees and, hence, stay longer as saplings in the understory, which may further increase their abundance over time. Contrary to expectations, none of the traits affected the abundance of liana saplings. This is surprising, as we included traits belonging to the leaf economics spectrum and stem economics spectrum, which are thought to be generally important for plant strategies and functioning, and therefore also for different life forms (Wright et al. 2004). It is likely that liana saplings in these forests are neither water limited (because of sufficient water availability) nor light limited, possibly because many liana species are light demanding and regenerate in open habitats (Schnitzer and Bongers 2002). Since traits are more strongly related to the regeneration niche than to the adult niche (Poorter 2007), the relationship between traits and abundance may be absent for lianas at the adult stage too. Instead of the importance of local plant abundance, traits may better explain species distributions and their presence along gradients of resource availability. For example, the importance of conservative traits for trees but not lianas would indicate that trees have an advantage under some limiting resources (e.g., wet forests with low light availability), whereas lianas would favor areas with high light availability (Schnitzer 2005).

## Conclusions

We evaluated the trade‐off between hydraulic efficiency and hydraulic safety, the associations among other relevant morphological and physiological traits, and the effect of traits on abundance for saplings of 51 tree and liana species. Trees showed the expected trade‐off between efficiency and safety, but lianas did not, indicating that safety and efficiency of lianas are partly controlled by different mechanisms than safety and efficiency of trees. This uncoupling of efficiency and safety for lianas could allow them to transport more water and potentially enhance their growth rates while not reducing their resistance to cavitation, which could potentially explain their success in drier forests. Conservative traits were positively related with abundance of tree saplings, probably because they enhance shade tolerance. However, none of the traits were related with the abundance of liana saplings, suggesting that other environmental factors limit liana abundance. Further studies are needed to underpin the mechanisms of the decoupling between efficiency and safety for lianas (self‐supporting vs. structural parasites) and the consequences of these differences for species performance and abundance.

## Data Availability

Data are available from the DANS repository: https://doi.org/10.17026/dans-xyg-4byf
