# Supplementary material for: The hydraulic efficiency–safety trade‐off differs between lianas and trees
Source: Ecology. 2019 Apr 8;100(5):e02666. doi: 10.1002/ecy.2666 (PMC6850011; doi:10.1002/ecy.2666)
Supplement: Supplementary file 5 [file ECY-100-na-s005.pdf]

Supporting Information. van der Sande, Masha T., Lourens Poorter, Stefan A. Schnitzer, Bettina M. J. Engelbrecht, Lars Markesteijn. 2019. The hydraulic efficiency–safety trade-off differs between lianas and trees. *Ecology*.

Appendix S5

Table S1: Differences in traits between lianas and trees tested using a t-test per trait, with the mean trait value per lifeform and the t-value and P-value of the t-test. All traits except for g_s were ln-transformed prior to analysis to meet the assumption of normality of the data. T-tests were performed using the *t.test* function, and normality tests using the *shapiro.test* function in R.

Trait	Mean lianas	Mean trees	t-value	P-value
Safety	1.12	1.81	-3.17	0.003
Efficiency	867.66	396.11	3.21	0.002
WD	0.45	0.44	0.11	0.916
MVL	78.40	62.97	1.64	0.108
Hv	0.016	0.021	-2.16	0.036
WUE	74.71	77.75	-0.87	0.389
SLA	170.50	169.16	0.07	0.944
LDMC	0.33	0.32	0.32	0.747
A_{area}	13.80	10.26	3.80	<0.001
g_s	0.19	0.14	4.34	<0.001
