# Supplementary material for: The hydraulic efficiency–safety trade‐off differs between lianas and trees
Source: Ecology. 2019 Apr 8;100(5):e02666. doi: 10.1002/ecy.2666 (PMC6850011; doi:10.1002/ecy.2666)
Supplement: Supplementary file 6 [file ECY-100-na-s006.pdf]

Supporting Information. van der Sande, Masha T., Lourens Poorter, Stefan A. Schnitzer, Bettina M. J. Engelbrecht, Lars Markesteijn. 2019. The hydraulic efficiency–safety trade-off differs between lianas and trees. *Ecology.*

Appendix S6

Table S1: Results of standardized major axis analyses to test how forest types differ in their relationships between hydraulic efficiency, safety and other stem and leaf traits. These analyses were done separately for lianas and trees ("Life form").

**Variable 1**	**Variable 2**	**Life form**	**P value**	**Test stat**	**Slope moist forest**	**Slope wet forest**
Hydraulic efficiency	Hydraulic safety	Liana	0.559	0.34	-0.001	-0.001
Hydraulic efficiency	Hydraulic safety	Tree	0.363	0.83	-0.005	-0.003
Hydraulic efficiency	WD	Liana	0.272	1.21	<0.001	<0.001
Hydraulic efficiency	WD	Tree	0.385	0.76	<0.001	<0.001
Hydraulic efficiency	MVL	Liana	0.372	0.80	0.043	0.032
Hydraulic efficiency	MVL	Tree	0.679	0.17	0.104	0.090
Hydraulic efficiency	Hv	Liana	0.003	9.13	<0.001	<0.001
Hydraulic efficiency	Hv	Tree	0.184	1.77	<0.001	<0.001
Hydraulic efficiency	WUE	Liana	0.413	0.67	0.011	0.015
Hydraulic efficiency	WUE	Tree	0.673	0.18	0.058	-0.070
Hydraulic efficiency	SLA	Liana	0.891	0.02	-0.042	0.040
Hydraulic efficiency	SLA	Tree	0.014	6.00	0.303	0.125
Hydraulic efficiency	LDMC	Liana	0.837	0.04	<0.001	<0.001
Hydraulic efficiency	LDMC	Tree	0.580	0.31	<0.001	<0.001
Hydraulic efficiency	A_{area}	Liana	0.409	0.68	0.004	0.004
Hydraulic efficiency	A_{area}	Tree	0.570	0.32	0.010	0.008
Hydraulic efficiency	g_s	Liana	0.384	0.76	<0.001	<0.001
Hydraulic efficiency	g_s	Tree	0.359	0.84	<0.001	<0.001
Hydraulic safety	WD	Liana	0.050	3.83	-0.281	0.150
Hydraulic safety	WD	Tree	0.049	3.87	0.071	0.128
Hydraulic safety	MVL	Liana	0.198	1.66	74.859	-43.505
Hydraulic safety	MVL	Tree	0.681	0.17	-22.266	-25.869
Hydraulic safety	Hv	Liana	0.003	8.92	-0.019	0.005
Hydraulic safety	Hv	Tree	0.851	0.04	-0.009	0.008
Hydraulic safety	WUE	Liana	0.899	0.02	-19.748	-20.851
Hydraulic safety	WUE	Tree	0.247	1.34	12.565	-20.067
Hydraulic safety	SLA	Liana	0.464	0.54	72.603	-54.448
Hydraulic safety	SLA	Tree	0.079	3.08	-65.093	-35.968
Hydraulic safety	LDMC	Liana	0.692	0.16	-0.099	0.085
Hydraulic safety	LDMC	Tree	0.817	0.05	0.047	0.051
Hydraulic safety	A_{area}	Liana	0.908	0.01	-6.347	-6.053
Hydraulic safety	A_{area}	Tree	0.778	0.08	-2.229	-2.442
Hydraulic safety	g_s	Liana	0.192	1.71	0.081	-0.046
Hydraulic safety	g_s	Tree	0.912	0.01	-0.035	-0.033
