# Supplementary material for: The hydraulic efficiency–safety trade‐off differs between lianas and trees
Source: Ecology. 2019 Apr 8;100(5):e02666. doi: 10.1002/ecy.2666 (PMC6850011; doi:10.1002/ecy.2666)
Supplement: Supplementary file 7 [file ECY-100-na-s007.pdf]

Supporting Information. van der Sande, Masha T., Lourens Poorter, Stefan A. Schnitzer, Bettina M. J. Engelbrecht, Lars Markesteijn. 2019. The hydraulic efficiency–safety trade-off differs between lianas and trees. *Ecology.*

Appendix S7

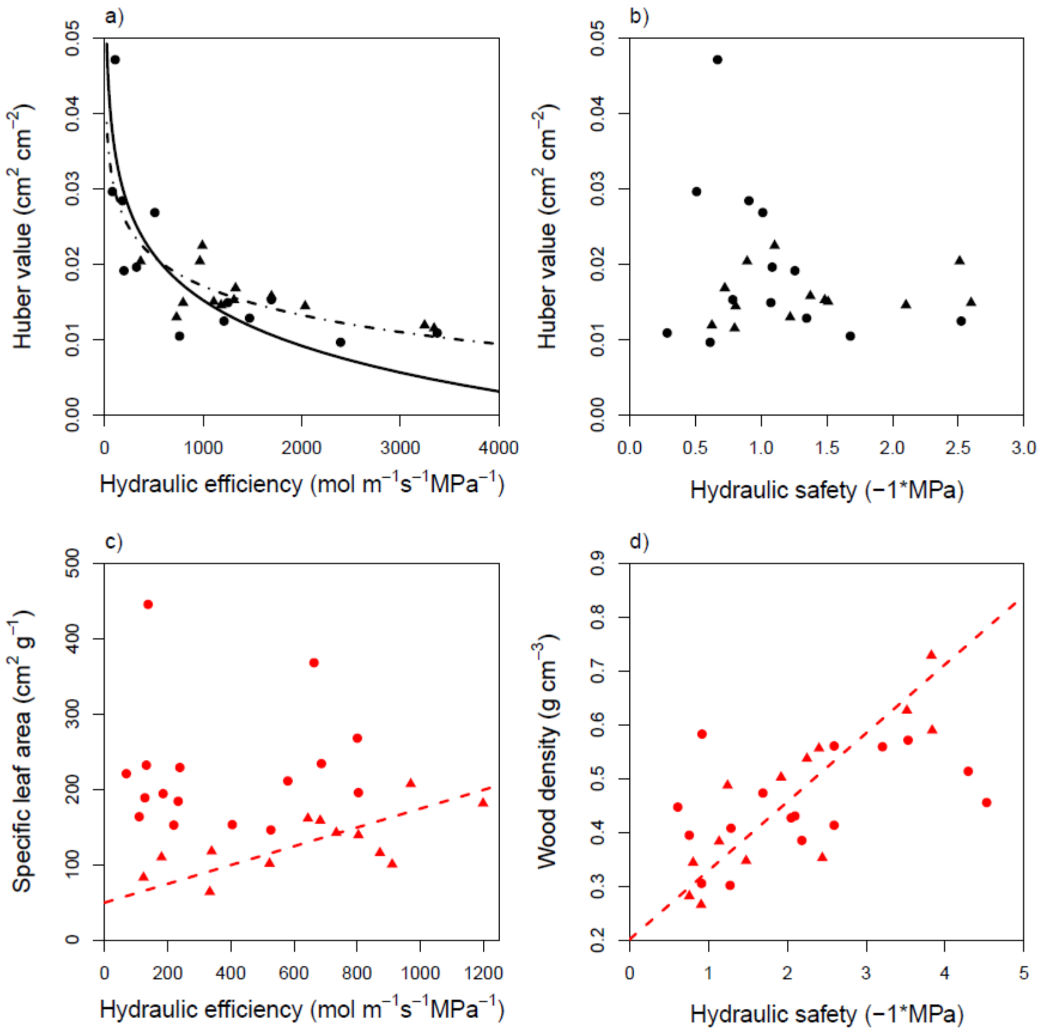

Figure S1: Relationships of hydraulic efficiency and hydraulic safety with traits that showed different slopes between forest types within the life form (see Appendix S1). Trees are given in red and lianas in black. Circles represent the moist forest and triangles the wet forest. Continuous lines are significant relationships for the moist forest, dashed lines are significant relationships for the wet forest, and no line indicates a non-significant relationship for that forest.
