# Supplementary material for: The hydraulic efficiency–safety trade‐off differs between lianas and trees
Source: Ecology. 2019 Apr 8;100(5):e02666. doi: 10.1002/ecy.2666 (PMC6850011; doi:10.1002/ecy.2666)
Supplement: Supplementary file 8 [file ECY-100-na-s008.pdf]

Supporting Information. van der Sande, Masha T., Lourens Poorter, Stefan A. Schnitzer, Bettina M. J. Engelbrecht, Lars Markesteijn. 2019. The hydraulic efficiency–safety trade-off differs between lianas and trees. *Ecology.*

Appendix S8

Table S1: Goodness-of-fit of the models evaluating the effect of traits (Predictor variable) and lifeform on abundance. Generalized linear models (GLMs) with Poisson error distribution and GLMs with negative binomial error distributions were tested. The goodness-of-fit is based on the residual deviance and degrees of freedom, using the pchisq function in R. We calculated the goodness-of-fit by 1-pchisq, so that values > 0.05 indicate a good fit of the model. In all cases, a GLM with negative binomial error distribution gave a good fit.

**Predictor variable**	**Poisson**	**Negative binomial**
Safety	<0.001	0.118
Efficiency	<0.001	0.120
WD	<0.001	0.121
MVL	<0.001	0.121
Hv	<0.001	0.119
WUE	<0.001	0.120
SLA	<0.001	0.122
LDMC	<0.001	0.120
A_{area}	<0.001	0.120
g_s	<0.001	0.118
