# Supplementary material for: The hydraulic efficiency–safety trade‐off differs between lianas and trees
Source: Ecology. 2019 Apr 8;100(5):e02666. doi: 10.1002/ecy.2666 (PMC6850011; doi:10.1002/ecy.2666)
Supplement: Supplementary file 9 [file ECY-100-na-s009.pdf]

Supporting Information. van der Sande, Masha T., Lourens Poorter, Stefan A. Schnitzer, Bettina M. J. Engelbrecht, Lars Markesteijn. 2019. The hydraulic efficiency–safety trade-off differs between lianas and trees. *Ecology.*

Appendix S9

Table S1: Results of t-tests to evaluate differences in traits between self-supporting lianas (N=5) and structural parasite lianas (N=5).

**Trait**	**t-value**	**df**	**p-value**
Hydraulic safety	0.73	6.64	0.493
Hydraulic efficiency	1.12	7.44	0.297
WD	-1.29	7.22	0.238
MVL	2.26	6.63	0.060
Hv	-1.12	4.80	0.314
WUE	1.76	6.35	0.126
SLA	-1.14	6.53	0.295
LDMC	-0.36	7.27	0.731
A_{area}	0.93	7.99	0.380
g_s	-0.01	6.15	0.996
