# Supplementary material for: The hydraulic efficiency–safety trade‐off differs between lianas and trees
Source: Ecology. 2019 Apr 8;100(5):e02666. doi: 10.1002/ecy.2666 (PMC6850011; doi:10.1002/ecy.2666)
Supplement: Supplementary file 10 [file ECY-100-na-s010.pdf]

Supporting Information. van der Sande, Masha T., Lourens Poorter, Stefan A. Schnitzer, Bettina M. J. Engelbrecht, Lars Markesteijn. 2019. The hydraulic efficiency–safety trade-off differs between lianas and trees. *Ecology.*

Appendix S10

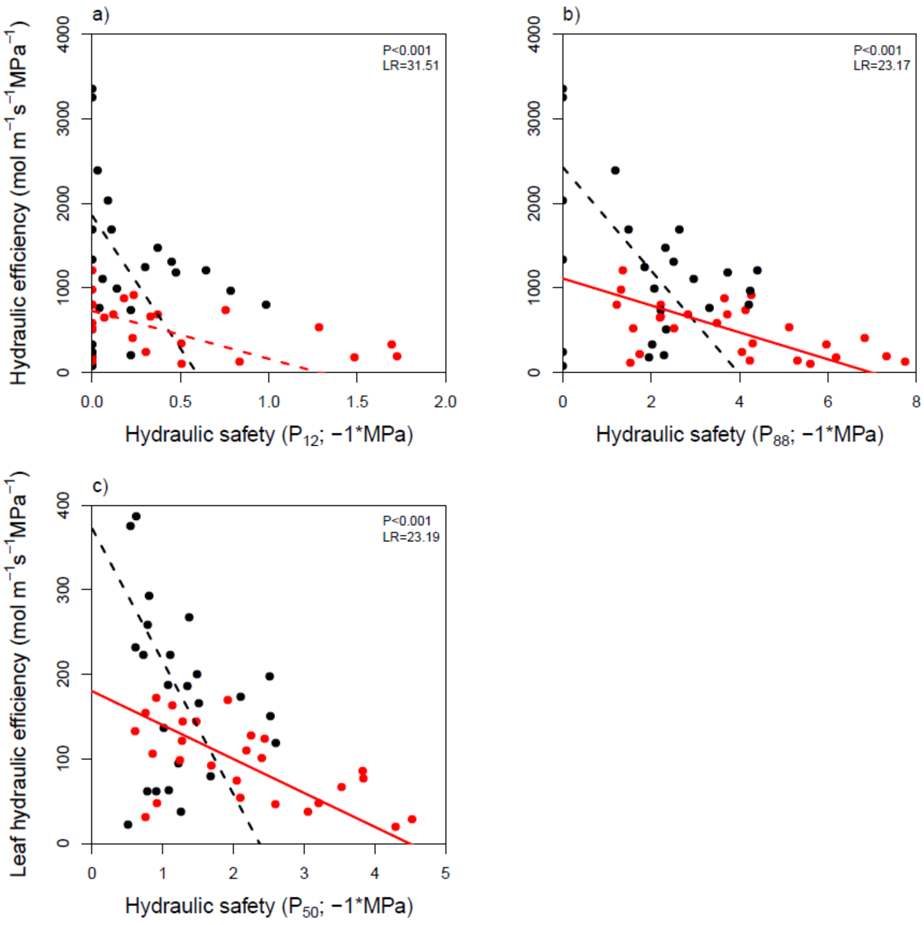

Figure S1: Relationship between sapwood-specific hydraulic efficiency (i.e. maximum sapwood hydraulic conductivity) and hydraulic safety calculated as the water potential at 12% (a) and 88% (b) loss of hydraulic conductivity multiplied by -1, and (c) the relationship between leaf-specific hydraulic conductivity and hydraulic safety (water potential at 50% loss of hydraulic conductivity multiplied by -1), for trees (red) and lianas (black). The lines represent the estimated relationship between the two variables (Table 1). Solid lines give significant relationships and dashed lines show insignificant trends. The likelihood-ratio test (LR) with corresponding significance (P) for the difference in slope between trees and lianas is given.
