# Supplementary material for: The hydraulic efficiency–safety trade‐off differs between lianas and trees
Source: Ecology. 2019 Apr 8;100(5):e02666. doi: 10.1002/ecy.2666 (PMC6850011; doi:10.1002/ecy.2666)
Supplement: Supplementary file 11 [file ECY-100-na-s011.pdf]

Supporting Information. van der Sande, Masha T., Lourens Poorter, Stefan A. Schnitzer, Bettina M. J. Engelbrecht, Lars Markesteijn. 2019. The hydraulic efficiency–safety trade-off differs between lianas and trees. *Ecology.*

Appendix S11

Table S1: Pearson correlation coefficients for trees (a) and lianas (b) corresponding the correlation networks in Fig. 1.

a) Trees	Hydraulic efficiency	Hydraulic safety	WD	MVL	Hv	WUE	SLA	LDMC	A_{area}
Hydraulic safety	-0.52								
WD	-0.47	0.61							
VL	0.28	-0.37	-0.59						
Hv	-0.62	0.14	0.16	-0.19					
WUE	-0.13	0.04	-0.05	-0.09	-0.1				
SLA	-0.07	-0.23	-0.47	0.33	0.14	0.14			
LDMC	-0.04	0.41	0.48	-0.43	-0.13	-0.22	-0.81		
A_{area}	0.44	-0.64	-0.49	0.27	-0.08	-0.04	0.38	-0.41	
g_s	0.4	-0.44	-0.32	0.27	-0.04	-0.63	0.19	-0.17	0.78

b) Lianas	Hydraulic efficiency	Hydraulic safety	WD	MVL	Hv	WUE	SLA	LDMC	A_{area}
Hydraulic safety	-0.25								
WD	-0.55	0.11							
MVL	0.62	-0.18	-0.59						
Hv	-0.66	-0.22	0.52	-0.66					
WUE	0.54	-0.2	-0.52	0.34	-0.23				
SLA	-0.32	-0.36	-0.02	-0.05	0.21	0.09			
LDMC	-0.08	0.4	0.38	-0.44	0.05	-0.19	-0.64		
A_{area}	0.86	-0.16	-0.55	0.57	-0.58	0.49	-0.4	0.06	
g_s	0.62	-0.02	-0.32	0.38	-0.48	-0.07	-0.57	0.22	0.82
