# Supplementary material for: The hydraulic efficiency–safety trade‐off differs between lianas and trees
Source: Ecology. 2019 Apr 8;100(5):e02666. doi: 10.1002/ecy.2666 (PMC6850011; doi:10.1002/ecy.2666)
Supplement: Supplementary file 12 [file ECY-100-na-s012.pdf]

Supporting Information. van der Sande, Masha T., Lourens Poorter, Stefan A. Schnitzer, Bettina M. J. Engelbrecht, Lars Markesteijn. 2019. The hydraulic efficiency–safety trade-off differs between lianas and trees. *Ecology.*

Appendix S12

Table S1: Results of standardized major axis analyses to test how trees and lianas differ in their relationships between hydraulic safety and wood density (WD), leaf dry matter content (LDMC), photosynthetic capacity (A_{area}), and stomatal conducntance (g_s). The analyses differ in those from Table 1 because a similar range in hydraulic safety values was used for trees and lianas (0-3 MPA*-1). N = 20 tree species and 24 liana species. The slope, P-value and R^2 is given per life form, as well as the likelihood-ratio (LR) test for the difference in slope between trees and lianas.

		**Trees**			**Lianas**			**Differences in slope of trees vs lianas**	
**Var 1**	**Var 2**	**Slope**	**P value**	R^2	**Slope**	**P value**	R^2	**LR**	**P-value**
Hydraulic safety	WD	0.14	0.165	0.10	0.21	0.619	0.01	1.98	0.160
Hydraulic safety	LDMC	0.11	0.238	0.08	0.12	0.054	0.16	0.22	0.642
Hydraulic safety	A_{area}	-4.45	0.160	0.11	-8.37	0.343	0.04	4.08	0.043
Hydraulic safety	g_s	-0.05	0.630	0.01	-0.07	0.925	0.00	1.08	0.298
