# Supplementary material for: The hydraulic efficiency–safety trade‐off differs between lianas and trees
Source: Ecology. 2019 Apr 8;100(5):e02666. doi: 10.1002/ecy.2666 (PMC6850011; doi:10.1002/ecy.2666)
Supplement: Supplementary file 13 [file ECY-100-na-s013.pdf]

**Supporting Information.** van der Sande, Masha T., Lourens Poorter, Stefan A. Schnitzer, Bettina M. J. Engelbrecht, Lars Markesteijn. 2019. The hydraulic efficiency–safety trade-off differs between lianas and trees. *Ecology.*

**Appendix S13**

**Table S1:** Correlations between hydraulic efficiency and other traits: wood density (WD), maximum vessel length (MVL), Huber value (Hv), water use efficiency (WUE), specific leaf area (SLA), leaf dry matter content (LDMC), photosynthetic efficiency ($A_{area}$), and stomatal conductance ($g_s$). Correlations are determined at species level (i.e. corresponding with Appendix S11 and Fig. 1b,c) and at individual level using two approaches: 1) standardizing the traits by subtracting the species mean trait value from the trait value of individual plants, and 2) bootstrapping our data by randomly pulling one individual of each species, calculated the correlations (i.e. based on 51 individuals), and performed this procedure a 1000 times to create 95% confidence intervals. Using the first approach, we remove the differences in average trait value among species, and we test for correlations within species (instead of testing for correlations among species). Using the second approach, we keep variation in trait values and make use of all data, but we do not test for correlations within species, only among species. The correlation strength (r) and p-value are given for the species-level correlations and the individual level correlations based on standardized variables. For individual level correlations based on bootstraps, the lower (2.5%) and upper (97.5%) confidence intervals are given.

| | | Species-level correlations | | Individual level correlations based on bootstraps | | | Individual level correlations based on standardized variables | |
|---|---|---|---|---|---|---|---|---|
| **Life form** | **trait** | **r** | **p-value** | **r** | **lowCI** | **upCI** | **r** | **p-value** |
| Lianas | WD | **-0.65** | 0.005 | **-0.54** | -0.66 | -0.39 | -0.17 | 0.058 |
| Trees | WD | **-0.44** | 0.013 | **-0.45** | -0.60 | -0.28 | 0.15 | 0.083 |
| Lianas | MVL | **0.71** | 0.000 | **0.55** | 0.42 | 0.69 | -0.03 | 0.729 |
| Trees | MVL | 0.29 | 0.156 | **0.26** | 0.10 | 0.43 | 0.04 | 0.597 |
| Lianas | Hv | **-0.65** | 0.001 | **-0.60** | -0.67 | -0.53 | **-0.32** | 0.000 |
| Trees | Hv | **-0.57** | 0.001 | **-0.59** | -0.68 | -0.51 | 0.07 | 0.404 |
| Lianas | WUE | **0.49** | 0.007 | **0.44** | 0.10 | 0.67 | -0.14 | 0.110 |
| Trees | WUE | -0.15 | 0.509 | -0.03 | -0.27 | 0.23 | **0.58** | 0.000 |

| | | | | | | | | |
|---|---|---|---|---|---|---|---|---|
| Lianas | SLA | -0.36 | 0.127 | **-0.30** | -0.43 | -0.15 | -0.05 | 0.572 |
| Trees | SLA | 0.00 | 0.741 | -0.02 | -0.20 | 0.23 | **-0.27** | 0.002 |
| Lianas | LDMC | -0.15 | 0.703 | -0.06 | -0.18 | 0.06 | -0.09 | 0.343 |
| Trees | LDMC | -0.09 | 0.825 | -0.05 | -0.28 | 0.14 | **0.48** | 0.000 |
| Lianas | $A_{area}$ | **0.89** | 0.000 | **0.81** | 0.70 | 0.89 | 0.03 | 0.721 |
| Trees | $A_{area}$ | 0.45 | 0.021 | **0.46** | 0.29 | 0.63 | **0.60** | 0.000 |
| Lianas | $g_s$ | **0.65** | 0.001 | **0.54** | 0.33 | 0.72 | 0.16 | 0.080 |
| Trees | $g_s$ | **0.42** | 0.036 | **0.40** | 0.23 | 0.56 | 0.01 | 0.930 |

*Notes:* These results show that species-level and bootstrapping at individual level show very similar correlation strength and direction, and that individual level correlations based on standardized variables show much weaker and sometimes different correlations compared to species-level. Unfortunately, we had a low number of individual per species and low variation in trait values among individuals per species, which may explain the weak correlation results. The similarities between correlations among species measured at the species level (i.e. original analyses) and correlations among species measured at the individual level (i.e. the bootstrapping) mainly indicates that individual-level trait values are overall a good estimation of species-mean trait values, and therefore result in similar trait correlations.
