# Supplementary material for: The hydraulic efficiency–safety trade‐off differs between lianas and trees
Source: Ecology. 2019 Apr 8;100(5):e02666. doi: 10.1002/ecy.2666 (PMC6850011; doi:10.1002/ecy.2666)
Supplement: Supplementary file 14 [file ECY-100-na-s014.pdf]

Supporting Information. van der Sande, Masha T., Lourens Poorter, Stefan A. Schnitzer, Bettina M. J. Engelbrecht, Lars Markesteijn. 2019. The hydraulic efficiency–safety trade-off differs between lianas and trees. *Ecology.*

Appendix S14

Table S1: Results of generalized linear models to test the relationship between traits ('predictor variable') and species' abundance. The slope, standard error (SE), z-value, and p-value are given. The significant relationships are shown in Fig. 6.

Predictor variable	Life form	Estimate of slope	SE	z value	p-value
Hydraulic safety	Lianas	0.10	0.63	0.17	0.868
	Trees	0.16	0.32	0.49	0.623
Hydraulic efficiency	Lianas	0.0007	0.00	1.65	0.099
	Trees	0.0004	0.00	0.38	0.707
WD	Lianas	-2.57	2.77	-0.93	0.354
	Trees	9.30	3.13	2.97	0.003
MVL	Lianas	-0.004	0.01	-0.39	0.695
	Trees	-0.033	0.01	-2.78	0.005
Hv	Lianas	-90.72	60.21	-1.51	0.132
	Trees	-13.84	36.10	-0.38	0.702
WUE	Lianas	0.05	0.04	1.45	0.148
	Trees	0.00	0.02	-0.06	0.948
SLA	Lianas	-0.010	0.01	-1.69	0.091
	Trees	-0.012	0.00	-2.75	0.006
LDMC	Lianas	2.10	4.76	0.44	0.659
	Trees	11.65	5.08	2.29	0.022
A_{area}	Lianas	0.17	0.10	1.74	0.083
	Trees	-0.12	0.15	-0.85	0.394
g_s	Lianas	0.34	8.59	0.04	0.969
	Trees	-1.39	9.03	-0.15	0.877
