# Supplementary material for: The hydraulic efficiency–safety trade‐off differs between lianas and trees
Source: Ecology. 2019 Apr 8;100(5):e02666. doi: 10.1002/ecy.2666 (PMC6850011; doi:10.1002/ecy.2666)
Supplement: Supplementary file 15 [file ECY-100-na-s015.pdf]

Supporting Information. van der Sande, Masha T., Lourens Poorter, Stefan A. Schnitzer, Bettina M. J. Engelbrecht, Lars Markesteijn. 2019. The hydraulic efficiency–safety trade-off differs between lianas and trees. *Ecology.*

Appendix S15

Table S1: Average absolute correlation strength of each trait with respect to all other traits, for lianas and trees separately.

**Trait**	**Lianas**	**Trees**
Hydraulic safety	0.29	0.34
Hydraulic efficiency	0.55	0.30
WD	0.46	0.37
Hv	0.46	0.16
MVL	0.48	0.28
WUE	0.37	0.14
SLA	0.37	0.28
LDMC	0.35	0.31
A_{area}	0.55	0.35
g_s	0.45	0.32
